# The weight-adjusted-waist index predicts all-cause and cardiovascular mortality in hypertension

**DOI:** 10.3389/fcvm.2025.1501551

**Published:** 2025-02-10

**Authors:** Yu Zheng, Zixing Nie, Yifan Zhang, Tao Sun

**Affiliations:** ^1^ The First Clinical College of Traditional Chinese Medicine, Hunan University of Chinese Medicine, Changsha, Hunan, China; ^2^ Department of Cardiology, The First Affiliated Hospital of Hunan University of Chinese Medicine, Changsha, Hunan, China

**Keywords:** weight-adjusted-waist index, hypertension, obesity, NHANES, mortality

## Abstract

**Background:**

Weight-adjusted-waist (WWI) is a novel indicator of obesity that reflects the degree of central obesity in the human body.

**Objectives:**

The study aimed to explore the relationship between WWI and mortality in hypertensive individuals.

**Methods:**

Cross-sectional data from the 2001–2018 National Health and Nutrition Examination Survey (NHANES) dataset were used in this study. The relationship between WWI and mortality was assessed using a weighted Cox proportional risk model; the nonlinear relationship was explored using restricted cubic splines. The robustness of the results was verified by subgroup and sensitivity analyses.

**Results:**

A cohort of 11,556 people with a diagnosis of hypertension was included in this study. As a continuous variable, WWI was linked to higher rates of mortality from all-cause (HR = 1.23, 95% CI = 1.14, 1.33) and cardiovascular disease (CVD) (HR = 1.43, 95% CI = 1.23, 1.66) with hypertension in Model 3 adjusted for variables. Using WWI as a tertile categorical variable, individuals in the highest tertile had a 33% higher risk of all-cause death (HR = 1.33, 95% CI = 1.14, 1.56) and a 65% higher risk of CVD death (HR = 1.65, 95% CI = 1.19, 2.27) than individuals in the lowest tertile. According to the subgroup analysis, almost all groups showed a consistent positive correlation between WWI and mortality related to all-cause and CVD.

**Conclusion:**

In adults with hypertension, there is a positive association between WWI and all-cause and CVD mortality.

## Introduction

Hypertension is a prevalent chronic non-communicable illness globally, impacting about one-third of the population and resulting in 10.4 million fatalities annually, and has grown to be a serious and expensive public health issue (1, 2). More importantly, it is acknowledged that hypertension is the main factor responsible for significant unfavorable cardiovascular events (3). As a result, prompt identification and treatment of hypertension is crucial to advance the prognosis of individuals and reduce the worldwide incidence of cardiovascular diseases (CVDs) (4). Obesity is a significant risk factor for CVDs (5). Individuals with obesity have a sustained metabolic disorder, increasing the risk of coronary heart disease (CHD), stroke, and heart failure (HF) (6). Identifying obesity is essential for individuals with hypertension, as these two conditions often occur together in adults. Obese individuals are 3.5 times more likely to acquire hypertension, and increased fat storage is responsible for 60% of cases of hypertension (6). Obesity and higher mortality are strongly correlated in older adults with hypertension (7).

Waist circumference (WC) and body mass index (BMI) are often used to assess obesity. Nevertheless, BMI cannot be considered a reliable indicator for differentiating between muscle and fat in the body, as the concept of the “obesity paradox” has demonstrated (8). For instance, older persons classified as obese due to a high BMI had a decreased mortality risk, which may be linked to a protective influence of augmented muscle mass (9). Conversely, elderly people with a low BMI often have muscular atrophy and elevated visceral fat, correlating with an increased mortality risk (10). WC exhibits a robust correlation with abdominal adiposity and is significantly linked to cardiovascular disease (CVD) risk factors and mortality (11). However, due to its strong link with BMI, WC is challenging to use as a standalone measure of obesity (12). While inaccurately identifying obesity in the general population may not pose immediate health risks, neglecting obesity care for hypertension patients escalates adverse consequences.

The weight-adjusted-waist index (WWI) is a novel anthropometric measure of central obesity, derived by standardizing WC to body weight (13). WWI diminishes the association between WC and BMI while preserving its efficacy in predicting visceral fat (10). A higher WWI indicates a more significant amount of fat and a lower proportion of muscle (14). Research has shown that WWI may accurately forecast the incidence of cardiovascular metabolic disorders and mortality (13). Furthermore, WWI demonstrated a positive correlation with fat mass and a negative correlation with muscle mass in older adults, indicating that its predictive capability may be less constrained by the age of the population (15).

No study has examined the association between hypertension mortality and WWI. Given the significance of identifying mortality predictors in hypertensive patients for the prompt identification and mitigation of risk factors, we commenced this study to address this knowledge gap. Using National Health and Nutrition Examination Survey (NHANES) data from 2001 to 2018, this research examined the relationship between WWI and all-cause and CVD mortality in adults with hypertension.

## Methods

### Study population

The NHANES survey is a program that investigates the influence of nutritional status on health promotion and illness prevention among non-institutionalized residents in the United States. Data collected in this survey are nationally representative. The research methodology received approval from the National Centre for Health Statistics Research Ethics Review Board, and all participants signed an informed consent form.

**Figure 1:**
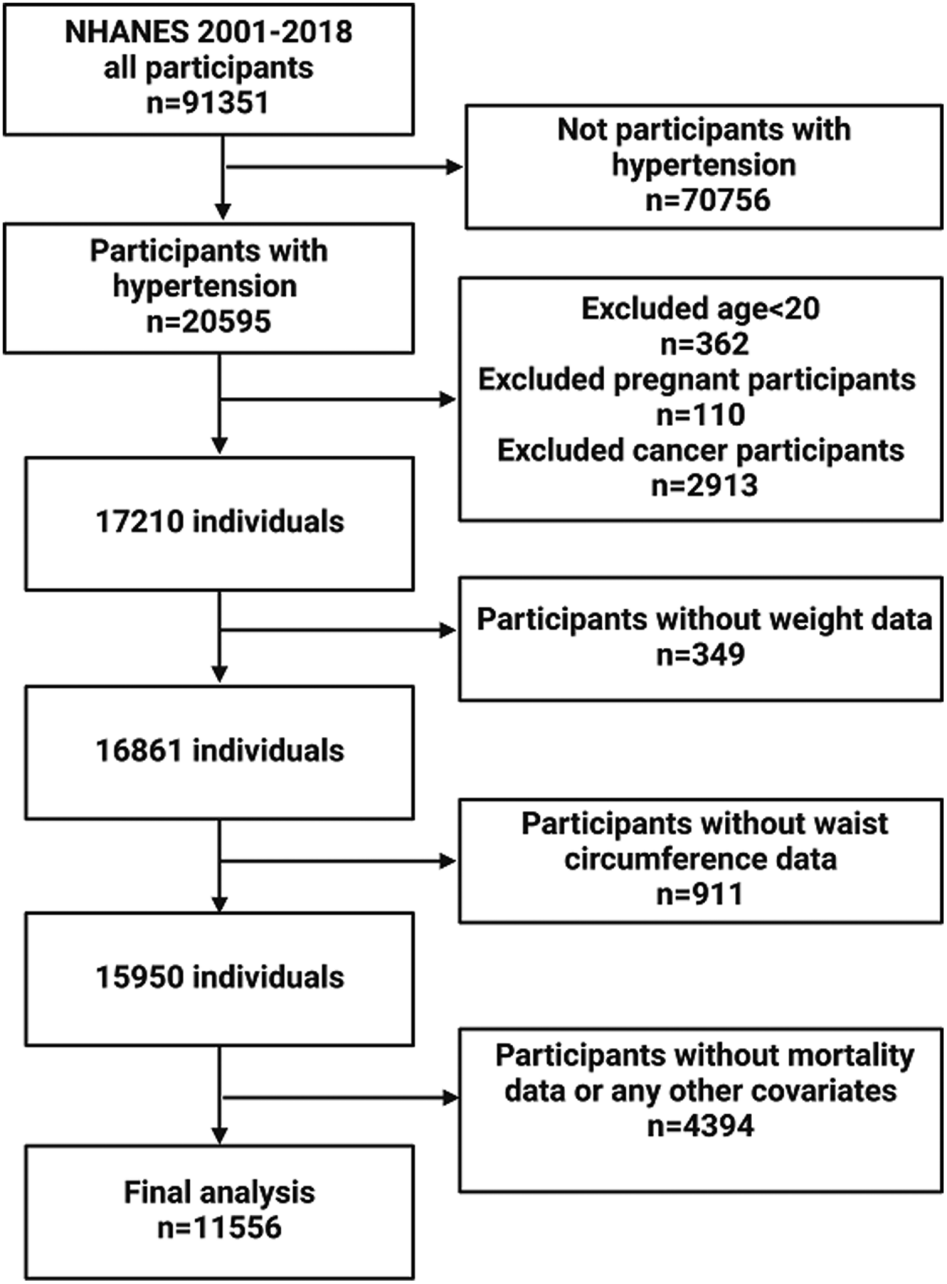
Flow chart of participants inclusion.

This research analyzed data from the NHANES database covering the period from 2001 to 2018 and included adults at least 20 years old with a diagnosis of hypertension. Hypertension was diagnosed by self-reported questionnaire data, antihypertensive medication, or a systolic or diastolic blood pressure reading of ≥140/90 mmHg to diagnose hypertension. Out of the 91,351 respondents, we removed individuals who were not hypertensive (*n* = 70,756), those under the age of 20 (*n* = 362), were pregnant (*n* = 110), cancer patients (*n* = 2,913), those with incomplete weight data (*n* = 349), those with incomplete WC data (*n* = 911), those with incomplete data on living status and other relevant factors (*n* = 4,394). As shown in Figure 1, 11,556 eligible individuals were ultimately included. The data utilized in this study were sourced from the publicly accessible website: https://www.cdc.gov/nchs/nhanes/.

### Weight-adjusted-waist index

WWI is an index for obesity that combines weight and WC. The WC and weight anthropometric measurements were obtained and documented by specialists from the NHANES program at the mobile checkpoints (13). WWI is calculated using the following formula: WWI = WC (cm) divided by weight (kg) square root (16). WWI was the exposure variable in our investigation.

### Ascertainment of mortality

We utilized the NHANES Public Use Linked Mortality File, which covers the time frame until December 31, 2019, to determine the survival of the participants. This research specifically examined the death rates related to all-cause and CVD. The International Classification of Diseases, Tenth Revision (ICD-10) criteria were followed in assigning codes I00 through I09, I11, I13, I20 through I51, and I60 through I69 to identify causes of death as CVD deaths.

### Assessment of covariates

The variables we considered as potential confounders were: age (year), gender (male/female), race/ethnicity (Mexican American/Other Hispanic/Non-Hispanic White/Non-Hispanic Black/Other race), education level (Below high school/High school or equivalent/Above high school), smoking status is defined by smoked at least 100 cigarettes in life (yes/no), drinking status is determined by alcoholic ≥4 drinks/day (yes/no), BMI (kg/m^2^), systolic blood pressure (SBP, mmHg), diastolic blood pressure (DBP, mmHg), CHD (n, number of subjects), congestive heart failure (CHF, n), angina (n), myocardial infarction (MI, n), stroke (n), and diabetes (n). The following laboratory parameters were obtained: alanine aminotransferase (ALT, U/L), aspartate aminotransferase (AST, U/L), uric acid (mg/dl), albumin (g/L), total cholesterol (TC, mmol/L), and high-density lipoprotein cholesterol (HDL-C, mmol/L). Definitions of CHF, CHD, angina, MI, and stroke were based on self-reported questionnaire data. Diabetes was diagnosed by self-reported questionnaire data or use of diabetes medications or hemoglobin A1C ≥6.5% or fasting blood glucose ≥126 mg/dl.

### Statistical analysis

Considering the intricate multi-stage sampling with probability design of NHANES, our analysis incorporated sample weights, clustering, and stratification. Regarding prior research (17), we transformed WWI into a categorical variable (tertiles). The baseline characteristics were compared across the WWI tertile groups. The differences between these tertiles were examined using chi-square tests for categorical data and *t*-tests for continuous ones. The mean with standard deviations (SD) was used to describe continuous data, while percentages were used to represent categorical variables. Three Cox regression models were developed to examine the correlation between WWI and mortality in adults with hypertension. Model 1 includes adjustments for age, sex, and race. Model 2 included additional adjustment variables for education, BMI, smoking status, and drinking status. Model 3 included additional variables for CHF, CHD, angina, MI, stroke, diabetes, uric acid, albumin, AST, ALT, HDL-C, TC, SBP, and DBP to Model 2.

We employed a restricted cubic spline (RCS) regression model, utilizing four nodes (5%, 35%, 65%, and 95%), to examine the possible non-linear relationship between WWI and mortality. This model was adjusted for the multivariable mentioned above. We undertook subgroup analyses by sex, age (<60 or ≥60), BMI (<30.00 or ≥30.00), CHF, CHD, angina, MI, and stroke. To ensure the reliability of the outcomes, sensitivity analyses were performed. Our study was conducted using R 4.3.2. A *P*-value of less than 0.05 was considered statistically significant.

## Results

### Baseline characteristics of participants

Our study involved a cohort of 11,556 adults diagnosed with hypertension with a mean age of 57.36 ± 14.99 years. Among them, 6,375 (55.2%) were men and 5,181 (44.8%) were females (Table 1). For all participants, the mean value of WWI was 11.30 ± 0.77 cm/√kg. The three tertiles of WWI are classified as follows: tertile 1: less than 10.96 cm/√kg, tertile 2: between 10.96 and 11.63 cm/√kg, and tertile 3: more than 11.63 cm/√kg. Patients in the highest tertile of WWI tended to be older, female, and were more likely to be of Mexican American, other Hispanic, Non-Hispanic White, and have lower levels of education than patients in the lowest tertile of WWI. Additionally, they had a higher prevalence of CHF, CHD, angina, MI, stroke, and diabetes. The mean (± SD) of values for BMI, WC, weight, uric acid, and SBP for those in WWI tertile 3 was higher than for the other tertiles; conversely, the mean (± SD) values for albumin, DBP, TC, and HDL-C in WWI tertile 3 were lower than those in the other tertiles.

**Table 1:** Basic characteristics of participants by weight-adjusted-waist index tertiles.

Characteristics	Weight-adjusted-waist index				*P-*value
	T1 (<10.96) *N* = 3,852	T2 (10.96–11.63) *N* = 3,852	T3 (>11.63) *N* = 3,852	Total *N* = 11,556	
Age	50.91 (14.93)	58.20 (14.07)	62.96 (13.40)	57.36 (14.99)	<0.01
Gender, *n* (%)					
Male	2534 (65.8)	2210 (57.4)	1631 (42.3)	6375 (55.2)	<0.01
Female	1318 (34.2)	1642 (42.6)	2221 (57.7)	5181 (44.8)	
Race/ethnicity, *n* (%)					
Mexican American	329 (8.5)	606 (15.7)	700 (18.2)	1635 (14.1)	<0.01
Other Hispanic	226 (5.9)	311 (8.1)	349 (9.1)	886 (7.7)	
Non-Hispanic White	1643 (42.7)	1649 (42.8)	1872 (48.6)	5164 (44.7)	
Non-Hispanic Black	1330 (34.5)	1007 (26.1)	733 (19.0)	3070 (26.6)	
Other race	324 (8.4)	279 (7.2)	198 (5.1)	801 (6.9)	
Education, *n* (%)					
Below high school	746 (19.4)	1035 (26.9)	1290 (33.5)	3071 (26.6)	<0.01
High school or equivalent	933 (24.2)	990 (25.7)	979 (25.4)	2902 (25.1)	
Above high school	2173 (56.4)	1827 (47.4)	1583 (41.1)	5583 (48.3)	
Smoked at least 100 cigarettes in life, *n* (%)					
Yes	2026 (52.6)	2175 (56.5)	2145 (55.7)	6346 (54.9)	<0.01
No	1826 (47.4)	1677 (43.5)	1707 (44.3)	5210 (45.1)	
Alcoholic ≥4 drinks/day, *n* (%)					
Yes	760 (19.7)	777 (20.2)	773 (20.1)	2310 (20.0)	0.88
No	3092 (80.3)	3075 (79.8)	3079 (79.9)	9246 (80.0)	
CHF, *n* (%)	121 (3.1)	178 (4.6)	311 (8.1)	610 (5.3)	<0.01
Angina, *n* (%)	116 (3.0)	160 (4.2)	267 (6.9)	543 (4.7)	<0.01
CHD, *n* (%)	161 (4.2)	267 (6.9)	372 (9.7)	800 (6.9)	<0.01
MI, *n* (%)	154 (4.0)	261 (6.8)	381 (9.9)	796 (6.9)	<0.01
Stroke, *n* (%)	137 (3.6)	222 (5.8)	299 (7.8)	658 (5.7)	<0.01
Diabetes, *n* (%)	524 (13.6)	1021 (26.5)	1459 (37.9)	3004 (26.0)	<0.01
BMI (kg/m2)	27.70 ± 5.60	30.79 ± 6.28	33.66 ± 7.43	30.72 ± 6.92	<0.01
Waist circumference (cm)	94.40 ± 12.22	105.02 ± 12.97	114.49 ± 15.58	104.64 ± 15.94	<0.01
Weight (kg)	82.50 ± 19.52	87.60 ± 21.57	90.67 ± 24.37	86.92 ± 22.17	<0.01
ALT (U/L)	26.35 ± 17.76	27.29 ± 20.89	25.00 ± 26.55	26.21 ± 22.06	<0.01
AST (U/L)	26.89 ± 18.98	27.19 ± 21.83	25.53 ± 16.37	26.54 ± 19.20	<0.01
Uric acid (mg/dl)	5.71 ± 1.47	5.87 ± 1.49	5.95 ± 1.55	5.85 ± 1.50	<0.01
Albumin (g/L)	42.74 ± 3.31	41.95 ± 3.25	41.15 ± 3.16	41.95 ± 3.30	<0.01
SBP (mmHg)	134.41 ± 19.16	136.59 ± 19.88	137.64 ± 20.76	136.21 ± 19.99	<0.01
DBP (mmHg)	77.33 ± 13.56	74.60 ± 13.93	71.54 ± 14.43	74.49 ± 14.18	<0.01
TC (mmol/L)	5.17 ± 1.12	5.12 ± 1.14	5.07 ± 1.14	5.12 ± 1.13	<0.01
HDL (mmol/L)	1.42 ± 0.46	1.33 ± 0.42	1.30 ± 0.40	1.35 ± 0.43	<0.01

Data are presented as mean ± SD or *n* (%); *n*, number of subjects; SD, standard deviation;%, weighted percentage; BMI, body mass index; CHF, congestive heart failure; CHD, coronary heart disease; MI, myocardial infarction; ALT, alanine aminotransferase; AST, aspartate aminotransferase; TC, total cholesterol; HDL-C, high-density lipoprotein cholesterol; SBP, systolic blood pressure; DBP, diastolic blood pressure.

### WWI and mortality outcomes

Our study had a mean follow-up period of 102.22 months and recorded an amount of 2,144 deaths from all-cause, with 615 deaths attributed to CVD (Table 2). After controlling for most possible confounders, the findings of Model 3 showed a positive relationship between WWI and mortality outcomes. In Table 2, we demonstrate the results of the three Cox regression models. Models 1, 2, and 3, showed a notable increase in all-cause mortality for each 1-unit increase in WWI. The HR (95% CI) were 1.18 (1.10, 1.26), 1.24 (1.15, 1.34), and 1.23 (1.14, 1.33) respectively. For CVD mortality, for each 1-unit increase in WWI, the HR (95% CI) were 1.29 (1.14, 1.45) for Model 1, 1.36 (1.17, 1.57) for Model 2, and 1.43 (1.23, 1.66) for Model 3. The *P*-values for mortality from all-cause and CVD were statistically significant in all three models.

**Table 2:** Weighted association between weight-adjusted-waist index and mortality.

	Weight-adjusted-waist index				*P*-trend
	Tertile 1 (<10.96)	Tertile 2 (10.96–11.63)	Tertile 3> (11.63)	Weight-adjusted-waist index (continuous)	
All-cause mortality					
Number of deaths/total	470/3,852	680/3,852	994/3,852	2,144/11,556	
Model 1 (HR 95% CI)	1.00	1.00 (0.84, 1.19)	1.27 (1.09, 1.48)	1.18 (1.10, 1.26)	<0.01
Model 2 (HR 95% CI)	1.00	1.04 (0.88, 1.24)	1.36 (1.15, 1.62)	1.24 (1.15, 1.34)	<0.01
Model 3 (HR 95% CI)	1.00	1.01 (0.86, 1.18)	1.33 (1.14, 1.56)	1.23 (1.14, 1.33)	<0.01
CVD mortality					
Number of deaths%)	127/3,852	205/3,852	283/3,852	615/11,556	
Model 1 (HR 95% CI)	1.00	1.05 (0.83, 1.33)	1.37 (1.05, 1.78)	1.29 (1.14, 1.45)	<0.01
Model 2 (HR 95% CI)	1.00	1.08 (0.85, 1.37)	1.44 (1.06, 1.94)	1.36 (1.17, 1.57)	<0.01
Model 3 (HR 95% CI)	1.00	1.16 (0.89, 1.52)	1.65 (1.19, 2.27)	1.43 (1.23, 1.66)	<0.01

Model 1: Adjusted for age, gender, race/ethnicity.

Model 2: Adjusted for age, gender, race/ethnicity, education, BMI, smoking status, drinking status.

Model 3: Adjusted for age, gender, race/ethnicity, education, BMI, smoking status, drinking status, diabetes, CHD, angina, MI, stroke, CHF, uric acid, albumin, AST, ALT, HDL-C, TC, SBP, DBP.

**Figure 2:**
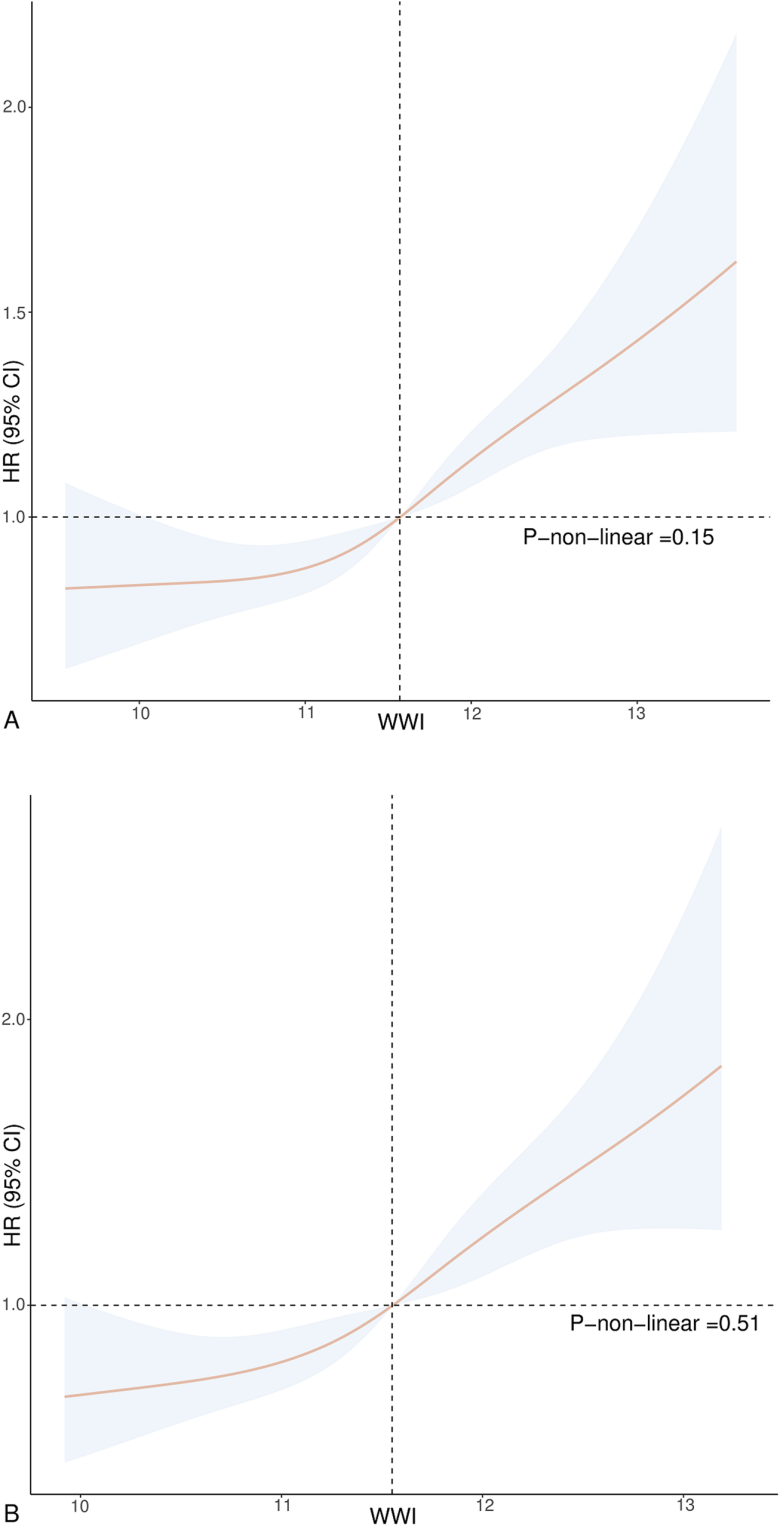
Association between WWI and all-cause **(A)** and CVD mortality **(B)** in hypertension. Adjusted for age, gender, race, education, BMI, smoking status, drinking status, diabetes, CHD, angina, MI, stroke, CHF, uric acid, albumin, AST, ALT, HDL-C, TC, SBP, and DBP. The solid and dotted lines represent the estimated values and their corresponding 95% CIs.

Participants in the highest tertile of WWI suffered a significantly elevated risk of all-cause and CVD mortality compared to those in the lowest tertile of WWI. The HR (95% CI) for all-cause mortality was 1.27 (1.09, 1.48) for Model 1, 1.36 (1.15, 1.62) for Model 2, and 1.33 (1.14, 1.56) for Model 3. The HR (95% CI) values for CVD mortality were 1.37 (1.05, 1.78) for Model 1, 1.44 (1.06, 1.94) for Model 2, and 1.65 (1.19, 2.27) for Model 3. Figure 2 indicates the outcomes of the Cox regression model fitted using RCS. The findings suggest a positive linear relationship between WWI and death from all-cause and CVD (*P*-none-linear = 0.15, *P*-none-linear = 0.51).

### Subgroup and sensitivity analyses

**Figure 3:**
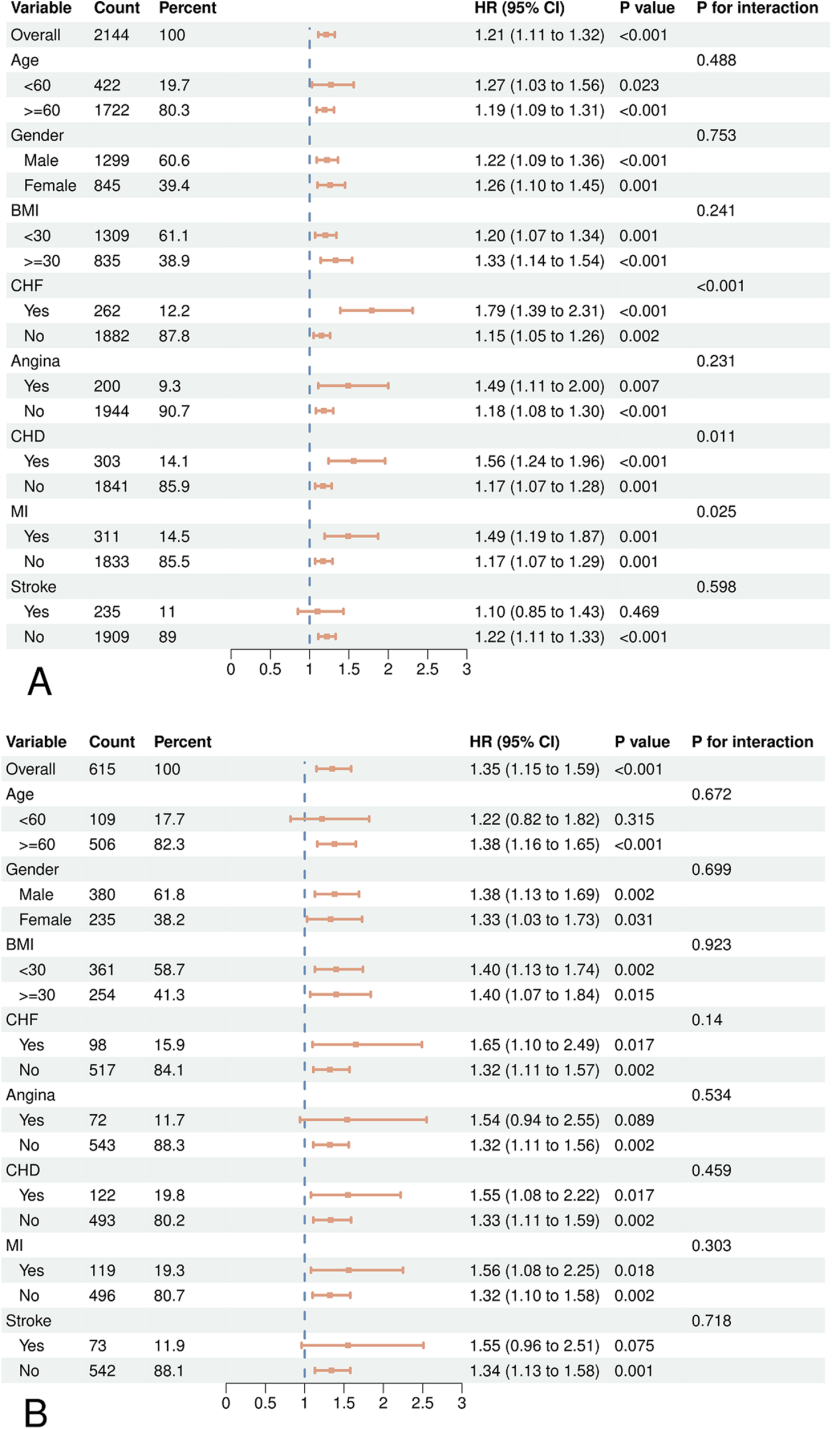
Forest plots of subgroup analyses of WWI and all-cause **(A)** and CVD **(B)** mortality in patients with hypertension. Age, gender, race, education, BMI, smoking status, drinking status, diabetes, CHD, angina, MI, stroke, CHF, uric acid, albumin, AST, ALT, HDL-C, TC, SBP, DBP were adjusted except the variable itself.

According to subgroup analysis, there was a greater risk of CVD and all-cause death in hypertension with increased WWI levels (Figure 3). However, no statistically significant variations were observed among specific subgroups regarding the association between CVD mortality and WWI, such as those under 60 years old (HR = 1.22, 95% CI = 0.82, 1.82), individuals with angina (HR = 1.54, 95% CI = 0.94, 2.55), and stroke populations (HR = 1.55, 95% CI = 0.96, 2.51). Among individuals who have had a stroke, the elevated WWI did not show a significant connection to heightened risks of mortality from all-cause (HR = 1.10, 95% CI = 0.85, 1.43). Furthermore, we observed an interaction between WWI and CHF, CHD, and MI in all-cause mortality.

The sensitivity analysis demonstrated that the results remained consistent, after excluding participants who died during the first two years of follow-up (Supplementary Table S1). The association between WWI and all-cause mortality remained consistent in hypertension, even when excluding individuals with CHF, CHD, and MI. Nevertheless, when patients with CHF, CHD, and MI were excluded, no significant correlation was observed between WWI as a categorical variable and CVD mortality (Supplementary Table S2).

## Discussion

This study included 11,556 hypertensive patients from the NHANES 2001–2018 cohort. We discovered a significant relationship between WWI from all-cause and CVD death in hypertension. Specifically, it was determined that increased WWI values were linked to an elevated risk of all-cause and CVD death. Similar outcomes were noted when the patients were segregated into groups based on age, gender, BMI, CHF, angina, CHD, MI, and stroke. Notably, in the first sensitivity analysis, the link between WWI and all-cause mortality was consistent with the overall participants. This suggests that our findings exhibit a certain level of robustness. The exclusion of patients with CHF, CHD, and MI had an impact on the association between CVD mortality and WWI. This finding indicates that the potential link between WWI and CVD mortality in hypertension may be primarily influenced by CHF, CHD, and MI. Based on the findings above, WWI can indicate mortality risk in hypertensive adults.

Previous research has investigated the connection between WWI and mortality risk across different populations. Research conducted in China revealed a positive connection between WWI and mortality from all-cause and CVD (18). A comparable connection can be observed among participants residing in the United States (19). Non-Asian people in the United States have a U-shaped association between WWI and death from all-cause. There is a correlation between extremely low and very high WWI values and an increased risk of death (16). Our results align with prior research, indicating a positive correlation between a higher level of WWI and elevated mortality risks. It is essential to emphasize that our study focused on hypertensive patients, revealing that in this cohort, WWI demonstrated a positive and linear association with CVD mortality and all-cause mortality, as opposed to a U-shaped association. Elevated WWI values correlate with adverse alterations in body composition, characterized by increased fat levels and decreased muscular mass (20). This suggests that hypertensive individuals may be more susceptible to the detrimental consequences of obesity, particularly elevated fat levels, in comparison to the overall healthy population. Conversely, reduced adiposity may mitigate the risk of elevated mortality in this population. Moreover, when eliminating patients with CHD, CHF, and MI, we noted a second tertile HR of less than 1 for WWI, although it was not statistically significant (Supplementary Table S2). This further indicates a significant correlation between obesity and CVD (21), with CVD being a primary cause of mortality in obese hypertensive individuals (6).

Obesity is the accumulation of excess fat in adipose tissue (22). Studies have consistently illustrated a direct link between obesity and the development of CVD risk, including hypertension, dyslipidemia, and type 2 diabetes (23), alongside a heightened vulnerability to CVD mortality (24). Individuals are considered obese if their BMI is 30 kg/m^2^ or higher (22). However, when examining individuals who are obese with a high BMI but without excess fat around the organs, to those with excess fat around the organs, the latter group has the highest risk of CVD, regardless of their BMI. Therefore, an excess of visceral or intra-abdominal fat might be a more crucial factor contributing to the onset of CVDs (25). These prior discoveries may explain the observed correlation in our investigation between higher WWI and increased prevalence of CHF, angina, CHD, MI, stroke, and diabetes in individuals with hypertension.

Obesity and hypertension are closely connected, with their interaction occurring via several processes. These mechanisms include activating the sympathetic nervous system, oxidative stress, endothelial dysfunction, and insulin resistance (7, 26). Specifically, those with a large amount of visceral fat have a notable decrease in the ability of arterial pressure receptors to regulate sympathetic activation (27). This leads to a highly active sympathetic nervous system. Sympathetic activation leads to both refractory hypertension and progressive cardiac injury (28, 29), which in turn induces myocardial remodeling and left ventricular remodeling, resulting in alterations to the form and function of the heart (30). Research demonstrates a notable rise in the stiffness of arteries and a substantial decline in their ability to comply and dilatation in individuals with obesity and hypertension (31). This modification of the structure of the arteries is strongly connected to the processes that lead to ischemic stroke and coronary artery disease (29). Furthermore, it dramatically affects the prognosis of hypertension in a detrimental way (32). The synergistic impact of obesity and hypertension is hazardous as it substantially augments the prevalence of CVDs and mortality (33). The subgroup analysis of all-cause mortality showed an interaction among the three CVD subgroups: CHD, CHF, and MI. Consistent with previous studies, the results suggest that obese hypertensive patients face a heightened danger of mortality, primarily due to CVDs (34, 35). In hypertensive patients, elevated WWI values are indicative of weakened health, marked by heightened visceral adipose tissue and related metabolic disruptions that amplify the likelihood of CVD mortality. After excluding patients with CHD, CHF, and MI from the analysis, it was found that the association between three tertiles of WWI and the risk of CVD death in hypertension was no longer statistically significant. Diseases such as CHD, CHF, and MI encompass the majority of CVDs, and they are often severe or even fatal. Therefore, it is reasonable to conclude that the absence of a significant relationship between WWI and CVD mortality is justifiable after excluding patients with all three of these aforementioned CVDs.

BMI is the prevailing physiological measure used to evaluate obesity status. One drawback of BMI is its failure to distinguish between adipose tissue and lean muscle mass, posing a challenge in accurately assessing body composition. Consequently, persons with greater muscle mass, such as athletes, may be erroneously categorized as obese owing to their higher BMI. Indeed, a considerable proportion of people may already have elevated body fat percentages, even if they are classified as non-obese according to BMI standards (36). In addition, the accuracy of BMI in categorizing obesity is affected by factors like age, gender, and race, which also restricts its use as an indicator of excessive or abnormal buildup of body fat (22). WC is a frequently used anthropometric measure that accurately indicates central obesity in humans and is strongly linked to the buildup of visceral fat deposits (37). While WC is valuable for evaluating the risk of obesity, it has limits when used alone (38), particularly in forecasting mortality risk in a population. Its effectiveness is mainly based on its conjunction with BMI (39). Hence, it is critical to formulate novel indicators to evaluate obesity status and its associated health risks precisely.

The WWI represents a novel approach to obesity assessment, incorporating both WC and body weight to provide a comprehensive evaluation. The index retains the benefits of WC measurement in indicating abdominal fat accumulation. Additionally, it weakens the potentially monolithic correlation that has traditionally existed between WC and BMI. WWI is considered a reliable measure of obesity along with BMI and WC (10, 14). Studies have shown that WWI has the ability to differentiate between muscle mass and fat mass (40), which may explain why the obesity paradox observed between BMI and mortality is not present in the association between WWI and mortality. WWI provides a thorough and exact method for evaluating obesity (13). In recent years, various studies have compared the predictive abilities of different anthropometric indices for mortality outcomes in participants. The findings indicate that WWI demonstrates superior predictive power for all-cause and CVD mortality, surpassing BMI and WC (13, 16). Additional metrics for assessing obesity encompass the waist-to-height ratio (WHtR) and a body shape index (ABSI). Research indicates that WHtR has a stronger correlation with CVD risk compared to BMI, although it shows a high correlation with BMI and a weaker correlation with mortality rates (41). In contrast, ABSI shows a weaker correlation with weight, height, and BMI, while demonstrating a stronger association with overall mortality risk (42). However, it is not as effective in predicting CVD mortality compared to WWI (13). In addition, various techniques, including bioimpedance analysis (BIA), dual-energy x-ray absorptiometry (DXA), and magnetic resonance imaging (MRI), can evaluate the distribution of adipose tissue within the body (43). However, these techniques can be complicated, require a significant amount of time, and come with a high price tag. In comparison, the measurement of WWI is both convenient and comprehensive, providing an adequate reflection of obesity status. By incorporating WWI into the health management of hypertension, it becomes possible to identify obese individuals and develop targeted health management strategies accurately. Managing obesity is crucial for enhancing the prognosis and minimizing the risk of complications in hypertension.

The study demonstrates several strengths. This is the first study of its kind to delve into the associations of WWI with both all-cause mortality and CVD mortality among community-dwelling hypertension patients within the United States. In addition, we utilized a meticulous multistage probability sampling design to analyze NHANES data, which improved the representativeness of our results. Finally, we considered various confounding factors to enhance the reliability and accuracy of our results.

Additionally, our study has some constraints. The blood pressure of participants was tested just once, perhaps leading to the misdiagnosis of hypertensive individuals. Other limitations include potential response bias and misclassification in the self-reporting of variables. Our study did not investigate the relationship between BMI and WC with all-cause mortality and CVD mortality in hypertensive patients. This comparison might enhance our evaluation of the predictive impact of several obesity indicators in hypertensive individuals. Given the nature of the NHANES database, a cross-sectional study, it is restricted to establishing a definitive causal connection between WWI and all-cause and CVD mortality in hypertension. Furthermore, the NHANES database sample has limitations in ethnic diversity, primarily focusing on the United States population. The study's findings are somewhat restricted in their generalizability to a broader population due to limitations in sample representativeness.

## Conclusion

The study revealed a positive association between heightened WWI values and an elevated risk of both all-cause and CVD mortality among hypertensive adults. These results indicate that WWI could be a straightforward and efficient indicator for forecasting mortality risk in these individuals. Future research can involve conducting extensive prospective cohort studies to validate these observations and delve deeper into the underlying biological mechanisms.

## Data Availability

The datasets presented in this study can be found in online repositories. The names of the repository/repositories and accession number(s) can be found below: https://www.cdc.gov/nchs/nhanes/index.htm.
