# Supplementary material for: The weight-adjusted-waist index predicts all-cause and cardiovascular mortality in hypertension
Source: Front Cardiovasc Med. 2025 Feb 10;12:1501551. doi: 10.3389/fcvm.2025.1501551 (PMC11847816; doi:10.3389/fcvm.2025.1501551)
Supplement: Supplementary file 1 [file Table1.pdf]

Supplementary Table 1. Hazard ratios (95% CI) of all-cause and CVD mortality according to WWI among patients with hypertension after excluding participants who died during the first two years of follow-up (n=10,521)

	**Weight-adjusted waist index**				***P***
	**Tertile 1 (< 10.96)**	**Tertile 2 (10.96–11.63)**	**Tertile 3 (> 11.63)**	**Weight-adjusted waist index (continuous)**	**trend**
**All-cause mortality**					
Number of deaths/total	415/3,569	609/3,520	843/3,432	1867/10,521	
Model 1	1.00	1.02 (0.85, 1.22)	1.24 (1.06, 1.44)	1.17 (1.09, 1.25)	<0.01
HR (95% CI) P-value					
Model 2	1.00	1.06 (0.89, 1.27)	1.33 (1.12, 1.58)	1.23 (1.14, 1.33)	<0.01
HR (95% CI) P-value					
Model 3	1.00	1.03 (0.88, 1.22)	1.31 (1.12, 1.53)	1.23 (1.14, 1.34)	<0.01
HR (95% CI) P-value					
**CVD mortality**					
Number of deaths (%)	110/3,569	185/3,520	242/3,432	537/10,521	
Model 1	1.00	1.12 (0.88, 1.42)	1.42 (1.09, 1.84)	1.31 (1.16, 1.49)	<0.01
HR (95% CI) P-value					
Model 2	1.00	1.14 (0.89, 1.46)	1.49 (1.10, 2.02)	1.38 (1.18, 1.61)	<0.01
HR (95% CI) P-value					
Model 3	1.00	1.20 (0.91, 1.58)	1.71 (1.23, 2.36)	1.48 (1.27, 1.73)	<0.01
HR (95% CI) P-value					

Model 1: Adjusted for age, gender, race/ethnicity

Model 2: Adjusted for age, gender, race/ethnicity, education, BMI, smoking status, drinking status

Model 3: Adjusted for age, gender, race/ethnicity, education, BMI, smoking status, drinking status, diabetes, CHD, angina, MI, stroke, CHF, uric acid, albumin, AST, ALT, HDL, TC, SBP, DBP
