# Supplementary material for: The weight-adjusted-waist index predicts all-cause and cardiovascular mortality in hypertension
Source: Front Cardiovasc Med. 2025 Feb 10;12:1501551. doi: 10.3389/fcvm.2025.1501551 (PMC11847816; doi:10.3389/fcvm.2025.1501551)
Supplement: Supplementary file 2 [file Table2.pdf]

Supplementary Table 2. Hazard ratios (95% CI) of all-cause and CVD mortality according to WWI among patients with hypertension after excluding participants with CHF, CHD, and MI (n=11,007)

	Weight-adjusted waist index				***P***
	**Tertile 1 (< 10.96)**	**Tertile 2 (10.96–11.63)**	**Tertile 3 (> 11.63)**	**Weight-adjusted waist index (continuous)**	**trend**
**All-cause mortality**					
Number of deaths/total	369/3,661	507/3,668	719/3,678	1595/11,007	
Model 1	1.00	0.909 (0.78, 1.06)	1.12 (0.98, 1.28)	1.11 (1.03, 1.20)	<0.01
HR (95% CI) P-value					
Model 2	1.00	0.952 (0.81, 1.12)	1.21 (1.05, 1.40)	1.18 (1.08, 1.28)	<0.01
HR (95% CI) P-value					
Model 3	1.00	0.928 (0.79, 1.09)	1.18 (1.01, 1.37)	1.18 (1.07, 1.30)	<0.01
HR (95% CI) P-value					
**CVD mortality**					
Number of deaths (%)	96/3,661	131/3,668	186/3,678	413/11,007	
Model 1	1.00	0.905 (0.64, 1.29)	1.17 (0.88, 1.56)	1.20 (1.03, 1.40)	0.02
HR (95% CI) P-value					
Model 2	1.00	0.912 (0.63, 1.32)	1.18 (0.84, 1.64)	1.23 (1.02, 1.48)	0.03
HR (95% CI) P-value					
Model 3	1.00	0.912 (0.63, 1.33)	1.15 (0.81, 1.64)	1.25 (1.03, 1.51)	0.02
HR (95% CI) P-value					

Model 1: Adjusted for age, gender, race/ethnicity

Model 2: Adjusted for age, gender, race/ethnicity, education, BMI, smoking status, drinking status

Model 3: Adjusted for age, gender, race/ethnicity, education, BMI, smoking status, drinking status, diabetes, angina, stroke, uric acid, albumin, AST, ALT, HDL, TC, SBP, DBP
